# The largest freshwater odontocete: A South Asian river dolphin relative from the proto-Amazonia

**DOI:** 10.1126/sciadv.adk6320

**Published:** 2024-03-20

**Authors:** Aldo Benites-Palomino, Gabriel Aguirre-Fernández, Patrice Baby, Diana Ochoa, Ali Altamirano, John J. Flynn, Marcelo R. Sánchez-Villagra, Julia V. Tejada, Christian de Muizon, Rodolfo Salas-Gismondi

**Affiliations:** ^1^ Department of Paleontology, University of Zurich, Karl-Schmid-Strasse 4, 8006 Zürich, Switzerland.; ^2^ Departamento de Paleontología de Vertebrados, Museo de Historia Natural-Universidad Nacional Mayor de San Marcos, Avenida Arenales 1256, Lima 11, Peru.; ^3^ Geosciences-Environnements Toulouse, Université de Toulouse, UPS (SVT-OMP), CNRS, IRD, 14 Avenue Édouard Belin, Toulouse 31400, France.; ^4^ Facultad de Ciencias e Ingienerías/Centro de Investigación para el Desarrollo Integral y Sostenible, Laboratorios de Investigación y Desarrollo, Universidad Peruana Cayetano Heredia, Lima, Peru.; ^5^ Departmento de Geología, Universidad de Salamanca, Salamanca 37008, Spain.; ^6^ Division of Paleontology, American Museum of Natural History, New York, NY 10024, USA.; ^7^ Department of Earth & Environmental Sciences, Columbia University, New York, NY 10027, USA.; ^8^ Graduate Programs in Biology and Earth and Environmental Sciences, The Graduate Center, City University of New York, New York, NY 10016, USA.; ^9^ Division of Geological and Planetary Sciences, California Institute of Technology, Pasadena, CA 91125, USA.; ^10^ Departement Origines et Evolution, CR2P UMR 7207, (MNHN, CNRS, UPMC, Sorbonne-Université), Muséum National d’Histoire Naturelle, rue Cuvier 57, 75005 Paris, France.

## Abstract

Several dolphin lineages have independently invaded freshwater systems. Among these, the evolution of the South Asian river dolphin *Platanista* and its relatives (Platanistidae) remains virtually unknown as fossils are scarce. Here, we describe *Pebanista yacuruna* gen. et sp. nov., a dolphin from the Miocene proto-Amazonia of Peru, recovered in phylogenies as the closest relative of *Platanista*. Morphological characters such as an elongated rostrum and large supraorbital crests, along with ecological interpretations, indicate that this odontocete was fully adapted to fresh waters. *Pebanista* constitutes the largest freshwater odontocete known, with an estimated body length of 3 meters, highlighting the ample resource availability and biotic diversity in the region, during the Early to Middle Miocene. The finding of *Pebanista* in proto-Amazonian layers attests that platanistids ventured into freshwater ecosystems not only in South Asia but also in South America, before the modern Amazon River dolphin, during a crucial moment for the Amazonian evolution.

## Teaser

A large river dolphin from Peruvian proto-Amazonia sheds light on the evolution of South Asian relatives.

## INTRODUCTION

**Fig. 1.**
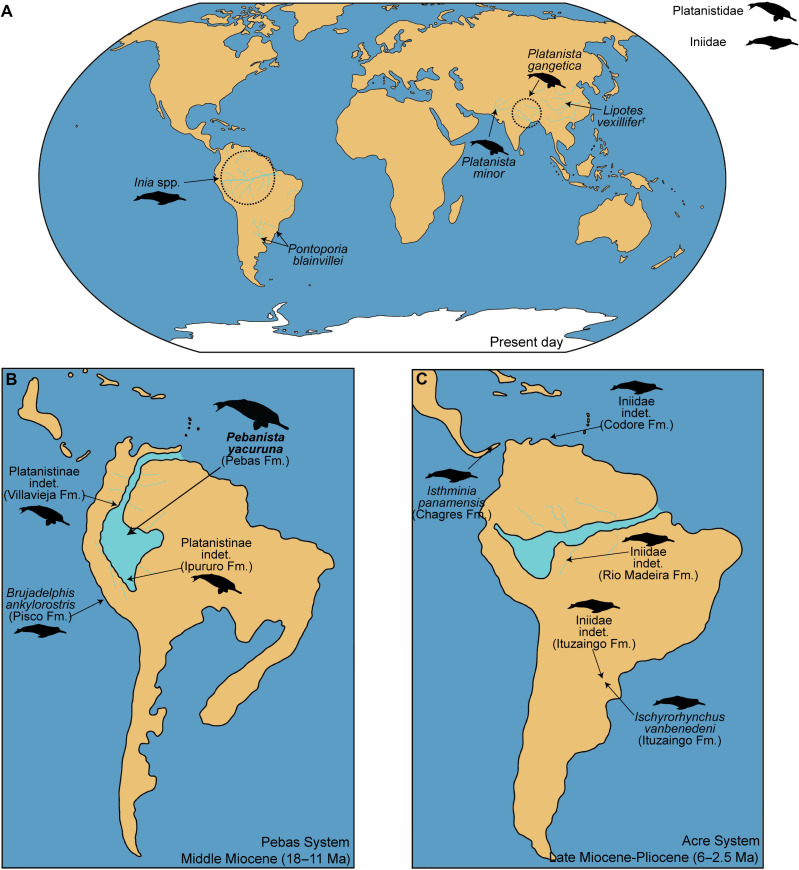
Biogeographical and paleobiogeographic distribution of Iniidae and Platanistidae through the Neogene. Schematic representation of the region highlighting the presence of epicontinental waters in South America (pale blue). Extant geographical ranges of the Amazon river dolphin *Inia* and the South Asian river dolphins *Platanista* (**A**). Distribution of fossil Iniidae/Platanistidae records in the Early to Middle Miocene Pebas System (**B**) and Late Miocene Acre System (**C**). Modified from Benites-Palomino *et al.* (*14*).

Cetacean freshwater transitions occurred in several areas asynchronously during the Neogene. Modern “river dolphins” arose from such events, as the similar morphology of these only distantly related taxa is the result of a clear convergent evolution (*1*, *2*). Among odontocetes (toothed cetaceans), four clades of river dolphins are recognized (Fig. 1A): Iniidae, Lipotidae, Platanistidae, and Pontoporiidae (*2*, *3*). The Yangtze river dolphin *Lipotes vexillifer* (Lipotidae) had fully riverine habits but was declared extinct a couple decades ago (*4*, *5*). Among the extant taxa, only *Platanista* (Platanistidae) and *Inia* (Iniidae) are strictly freshwater inhabitants (*6*), as the La Plata dolphin *Pontoporia blainvillei* (Pontoporiidae) roams shallow coastal waters.

*Platanista* from the South Asian river systems (Fig. 1) is one of the most enigmatic toothed cetaceans and unique by bearing enlarged, thin and pneumatic supraorbital crests that enclose the melon, a fatty structure integral to the echolocation system, which the animal uses to locate and capture prey in muddy waters. Echolocation in *Platanista* is so dominant that the animal is almost blind (*7*). The evolutionary history of *Platanista* (*8*) and kin remains elusive because fossil data of close relatives are restricted to marine forms such as *Araeodelphis*, *Pomatodelphis*, *Prepomatodelphis*, and *Zarhachis* (*2*, *3*). Contrarily, distant Platanistoidea relatives are one of the most diverse and frequently fossilized cetaceans, with records ranging from the Late Oligocene until the Middle Miocene. A similar situation pertains to the South American river dolphin *Inia* (Iniidae), whose fossil relatives have mostly been found in marine environments (*9*–*11*), with the exception of *Ischyrorhynchus* from the Late Miocene of Argentina (*12*). The overall fossil record of river dolphins is of limited value because the factors that led to repeated freshwater lifestyles from marine ancestors in Cetacea would preferably require fossils of freshwater forms (*13*, *14*).

Here, we describe a previously unknown platanistid dolphin found in Early to Middle Miocene layers of Peruvian Amazonia. Its holotype skull is characterized by a robust and long rostrum with enlarged teeth, well-developed supraorbital crests, a large temporal fossa, and a deep circumnarial basin. A series of phylogenetic analyses place the new taxon as a sister group to extant *Platanista*, thus demonstrating that at least two clades of odontocetes (Platanistidae and Iniidae) transitioned into freshwater environments in South America. Size estimations based on cranial measurements of the holotype of the new species and specimens referred to the same genus indicate that the new dolphin likely is the largest known freshwater odontocete, at 2.8 to 3.5 m at a minimum, surpassing the 2.5-m maximum size of modern “river” dolphins. Such a large body size, also recorded in other proto-Amazonia inhabitants (i.e. fishes and crocodilians), might be attributed to the large resource availability in proto-Amazonian ecosystems (*15*–*18*). Additional factors that may have contributed to the great body size of this new taxon include the lack of direct predators and competitors in the Pebas mega-wetland system. This finding confirms not only an independent marine-freshwater transition of cetaceans in South America but also that this diversity in the vast Pebas mega-wetland system might have greatly benefited from the warmer Middle Miocene climatic conditions in the area.

## RESULTS

### Systematic paleontology

Odontoceti

Platanistidae

*Pebanista yacuruna* sp. nov.

(Fig. 2).

**Fig. 2.**
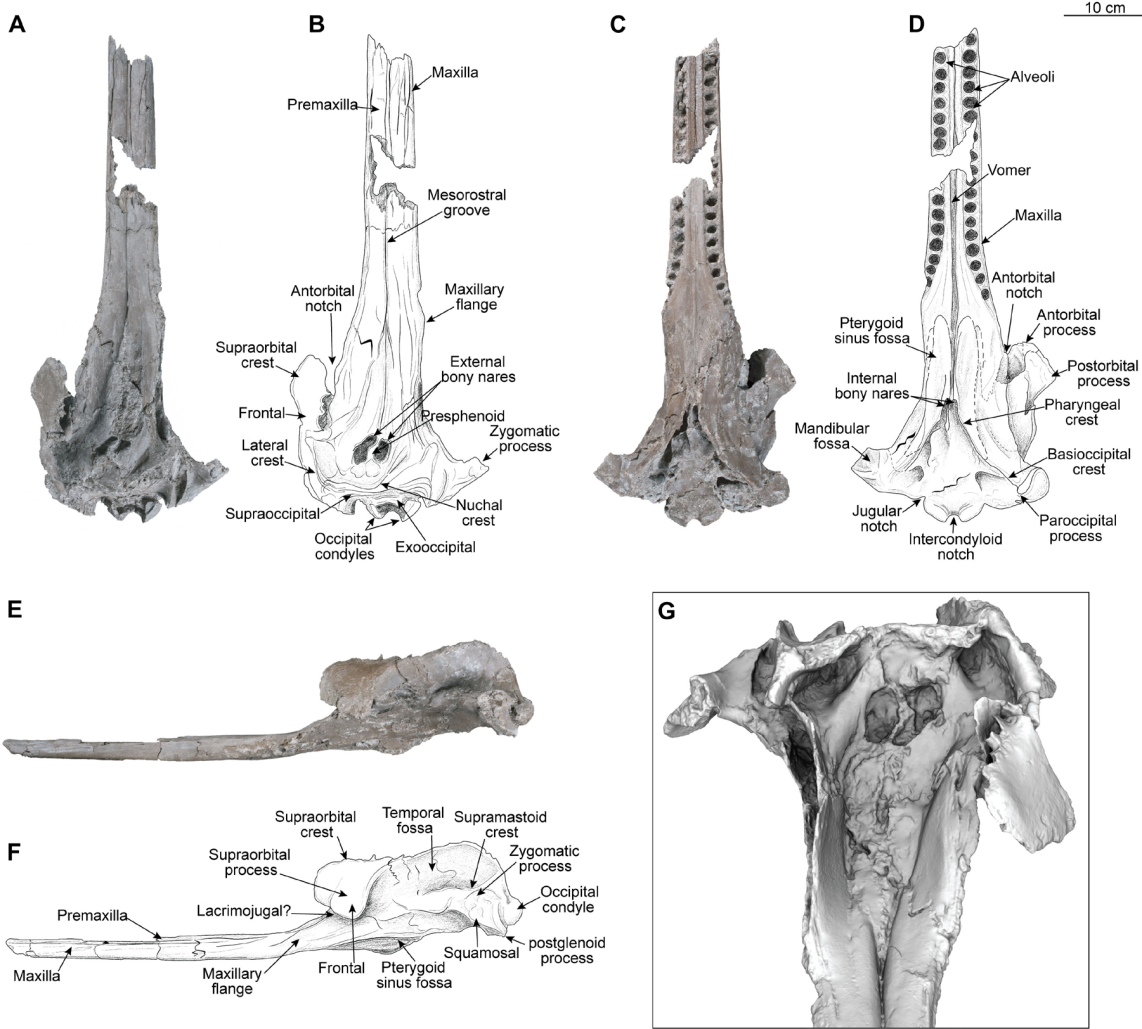
*Pebanista yacuruna* gen. et sp. nov., MUSM 4017. Holotype skull in dorsal (**A** and **B**), ventral (**C** and **D**), left lateral (**E** and **F**), and anterodorsal views (F and **G**).

### Etymology

The generic name *Pebanista* stresses the relationship between this taxon from the Pebas Fm. (section S1) and the extant Ganges and Indus river dolphins *Platanista* (*Platanista gangetica* and *Platanista minor*). The specific Kichua (northern Quechua) name honors the “yacuruna,” a mythical water creature in the Peruvian Amazonia.

### Holotype

MUSM 4017, an isolated skull from an adult individual that preserves the posterior part of the rostrum, facial region including part of the right supraorbital crest, the temporal and occipital regions (Fig. 2, section S2, and figs. S3 to S5). The specimen is permanently stored at the vertebrate palaeontology collection of the Museo de Historia Natural de la Universidad Nacional Mayor de San Marcos (MUSM).

### Locality, age and horizon

MUSM 4017 was collected in 2018 in stratigraphic levels that correspond to the upper Pebas Fm. exposed along the Rio Napo, Loreto, Peru (latitude, −3.012468°; longitude, −73.404855°). The palynological assemblage indicates freshwater environments, assignable to palynological Zones T-13 to Zone T-15 of Jaramillo *et al.* (*19*), ranging from the late Early Miocene to the Middle Miocene [circa 17 to 14 million years (Ma)]. Maximum likelihood analysis further constrains the age to the latest Early Miocene (circa 16.5 Ma.; section S1 and figs. S1 and S2).

### Diagnosis and remarks

The holotype skull of *Pebanista yacuruna*, MUSM 4017, has a preserved condylobasal length of 698 mm and an estimated bizygomatic width of 281 mm. The sutures between the cranial bones (e.g., maxilla-premaxilla suture along the rostrum) are well closed or fused, indicating an adult stage. *Pebanista* is recognized as a member of Platanistidae by having the vertex of the skull deviated leftwards (Fig. 2, A and B); asymmetry of the premaxillae in the rostrum and facial areas of the skull; braincase anteroposteriorly shorter than wide; and lack of contact of the palatines, with both projecting dorsolaterally (figs. S3 and S4). The rostrum of the holotype specimen is dorsoventrally flattened and elongated, a condition shared with the extinct *Pomatodelphis*, *Prepomatodelphis*, and *Zarhachis*, in contrast to the transversely compressed rostrum of extant *Platanista*. On the preserved portion, the rostrum is formed by the premaxillae, maxillae, and vomer, being much more transversely robust than in other platanistids. The rostrum exhibits several well-preserved dental alveoli; these are proportionally larger than those of other platanistids (fig. S4). The facial region of the skull exhibits a well-developed circumnarial basin, delimited laterally by the supraorbital crests and posteriorly by the nuchal crest. The external bony nares are displaced to the left, creating an asymmetric array of the surrounding bones. *Pebanista* displays greatly developed lateral supraorbital crests, projecting dorsomedially over the level of the facial region of the skull. These crests are formed by the frontal bone, unlike in *Pomatodelphis* and *Zarhachis* in which they are formed by the frontal laterally and the maxilla medially, and unlike in *Platanista* in which the crests are only formed by the maxillae. The supraorbital crests in *Pebanista* are robust but transversely flattened as in *Platanista*. The dorsomedial edge of the crests presents several large vacuities, which could foreshadow the full excavation of the crest in *Platanista*, which receives the dorsal extension of the pterygoid sinus (*20*). Only the left orbit is preserved, which in lateral view is proportionally shorter than other platanistids, a condition solely shared with extant *Platanista*. The pterygoids in *Pebanista* cover most of the palatines ventrally, except for a narrow lateral stripe. *Pebanista* differs from *Pomatodelphis* and *Zarhachis* by having transversely compressed walls of the supraorbital crests, partly resembling those of *Platanista*. The temporal fossa is anteroposteriorly longer than high and extends posteriorly into the occipital region (fig. S5). Posteriorly, the nuchal crest joins the supraorbital crest, giving the skull a squared posterior outline in dorsal view. The occipital shield projects slightly toward the anterior region of the skull, but it is not possible to assess whether this is its true shape, or a condition resulting from taphonomic compression.

### Additional materials

cf. *Pebanista* MUSM 3593 an isolated rostral fragment and Platanistidae indet. MUSM 4759 an isolated tympanic bulla (section S2 and fig. S6).

### Phylogenetic analyses

**Fig. 3.**
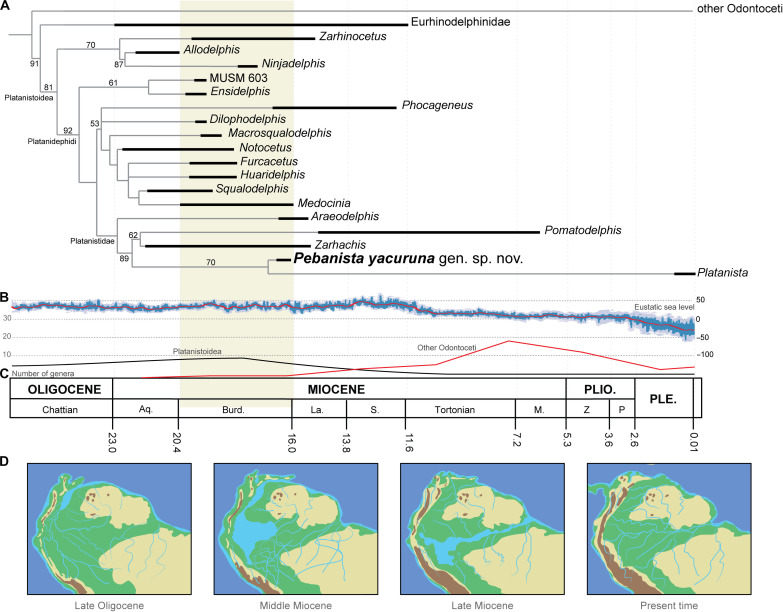
Phylogenetic relationships of *Pebanista yacuruna* gen. et sp. nov. and the evolutionary context of Platanistoidea within proto-Amazonia. Adams consensus from three most parsimonious trees (**A**); eustatic sea level evolution (*63*) across the mid-Late Neogene (**B**); diversity of Platanistoidea (*28*) versus other Odontoceti clades (**C**); geographical evolution (*49*, *50*) of the Neotropical region (**D**).

In all of our parsimony phylogenetic analyses, *Pebanista* was recovered within Platanistidae (Fig. 3 and fig. S7), with many of the other phylogenetic relationships being consistent with those of a prior work (*21*). All characters were equally weighted in a first analysis yielding phylogenies with poor intraclade resolution (low node support and high number of polytomies; section S2). Subsequent analyses were conducted with implied weighting of homoplasious characters (*22*, *23*), which resulted in improved support values for the two main Platanistoidea clades Platanistidae and Squalodelphinidae. Within Platanistidae, the two clades were supported with high bootstrap values (>70), one containing the fossil taxa *Zarhachis* and *Pomatodelphis* and the second uniting *Pebanista* and the extant *Platanista* (bootstrap value = 70). Following prior studies (*21*), and due to better resolution within Squalodephinidae, we opted for calculating the Adams consensus.

### Body size reconstruction

The estimated body size of the holotype specimen of *Pebanista yacuruna* is 280 cm, based solely on the bizygomatic width. The bizygomatic width of cf. *Pebanista* MUSM 3593 (estimated from the holotype specimen proportions) resulted in an estimated body length of 347 cm. However, prior works have shown that regression equations using the bizygomatic width might underestimate body lengths, especially for hyperlongirostrine taxa such as *Zarhachis* (*21*), instead suggesting the use of the condylobasal length of the skull, a feature not preserved in the holotype skull of *Pebanista*. Thus, the estimated body sizes for *Pebanista* (range, 281 to 347 cm) should be considered minima for this fossil platanistid dolphin.

## DISCUSSION

The South Asian river dolphins *Platanista gangetica* (Ganger river dolphin) and *P. minor* (Indus river dolphin) are the sole extant platanistids. Their fossil relatives, the marine Platanistoidea, were highly diverse between the Oligocene and the Early Miocene, reaching a cosmopolitan distribution. Their wide array of body sizes and skull morphologies indicates that they occupied different trophic levels and developed diverse predatory strategies (*21*, *24*, *25*). The fossil record indicates that the peak diversity of platanistoids was reached during the Early Miocene, a time of global cooling and increased subsidence in the Andean-Amazonian foreland basin system (*26*), including many records of squalodelphinids and allodelphinids (*27*), but began declining toward the Middle Miocene (*28*). Changes in sea level and other oceanic conditions related to the Middle Miocene Climatic Optimum, as well as the emergence of other toothed cetacean groups such as delphinoids (*29*, *30*), beaked whales (*31*), and physeteroids (*32*–*34*), may be related to the decline of platanistoids in marine environments (*35*). Such ecological displacement might have resulted in the surviving Platanistidae being restricted to freshwater environments.

The fossil record of Platanistidae is sparse. Some platanistoids from North America, such as *Araeodelphis* and *Dilophodelphis*, have been phylogenetically placed either within Platanistidae or Squalodelphinidae (*20*, *21*, *24*, *25*). Our phylogenetic analyses recovered *Araeodelphis*, a small taxon from the Early Miocene Calvert Fm. in Maryland (*36*), as the most basal stem member of the Platanistidae, in agreement with prior studies (*20*, *21*, *24*). Within Platanistidae, two clades are recognized: the first includes *Pomatodelphis* and *Zarhachis* (Fig. 3), both found in coastal marine environments from North America. These taxa are characterized by a long slender rostrum with small teeth, a minor development of the supraorbital crests, and a vertex mostly symmetrical when compared to *Platanista*. In addition, vertebrae found in Middle Miocene layers of Venezuela have been tentatively referred to *Zarhachis*, but the material is poorly diagnostic (*27*, *37*). Our phylogenetic analyses unambiguously recovered *Pebanista* as the sister taxon of *Platanista*, thus constituting the closest known relative of the extant South Asian river dolphins. *Pebanista* displays characters previously used to nest together *Pomatodelphis* and *Zarhachis*, such as the flattened rostrum and transverse expansion of the premaxilla (*38*, *39*). However, *Pebanista* also has numerous characters found in *Platanista*, including reduction of the orbit and strong asymmetry of the facial region, medially concave supraorbital crests, enlargement of the temporal fossae, and thickening of the zygomatic process of the squamosal (*40*). In addition, *Pebanista* also has enlarged teeth, which along with the robust rostrum and well-developed skeletal muscle insertions on the skull, it suggests an active raptorial feeding behavior (*41*). The supraorbital crests in *Pebanista* also are distinctive: These are more transversely robust than the thin plate-like crests of *Platanista* but not as robust as in *Zarhachis* or *Pomatodelphis*. Nevertheless, the inner structure of the crests indicates the presence of areas with higher bone density in the outer surface of the supraorbital crests and slightly lower densities medially. This might have facilitated focusing the sound waves of the biosonar system (*20*), also evidenced by the dorsomedial orientation of the supraorbital crests and their medially concave inner surface. Furthermore, the medial surface of the crests of *Pebanista* has a series of cavities or vacuities, resembling the condition observed in *Platanista*, which receives the dorsal extension of the pterygoid sinus (*38*, *39*).

Odontocetes invaded fresh waters several times independently during the Neogene (Fig. 1). Among the four extant clades of river dolphins, only *Inia*, *Platanista*, and the recently extinct *Lipotes* are restricted to freshwater environments, as *Pontoporia* also inhabits coastal environments of Brazil and Argentina. The marine-freshwater transition of Iniidae is better understood than that of Platanistidae because of new findings of the past decade. Two close relatives of *Inia* have been recovered in Late Miocene marine layers of Panama and Peru: *Brujadelphis* and *Isthminia*, respectively (*9*, *10*), indicating that iniids still inhabited coastal marine environments during those times. More recently a third iniid, *Kwanzacetus*, was recovered from rocks of the same age in Angola (*11*), further denoting the high diversity and broad geographic distribution of these cetaceans in marine environments. Marine iniids appear to have survived into the Pliocene, as evidenced by an isolated earbone from the Codore Fm. of Venezuela, which despite resembling that of extant *Inia*, still retained a cochlear morphology better suited for marine environments (*42*). There is little evidence of freshwater iniids in South America, but fossils from the Ituzaingo Fm. in Argentina, as the ones referred to the genus *Ischyrorhynchus*, already indicate the presence of iniids in fluvio-deltaic environments as early as the Late Miocene (*43*). The invasion of freshwater habitats thus must have occurred much earlier and independently in proto-Amazonia by *Pebanista* and in South Asia by *Platanista*. Previously described material from La Venta in Colombia and the Fitzcarrald Arch in Peru already provide evidence that platanistids invaded freshwater systems in South America during the Middle Miocene. Such findings comprise two isolated earbones respectively found in each locality. Both the La Venta and Fitzcarrald periotics display characters solely found in *Platanista* earbones, such as a great reduction of the posterior process and the reduced aperture for the cochlear aqueduct (*13*, *14*, *44*). Thus, the two morphotypes are much closer to *Platanista* than to *Zarhachis* or *Pomatodelphis* and as such might represent taxa closely related to the clade of *Pebanista* and *Platanista*.

**Fig. 4.**
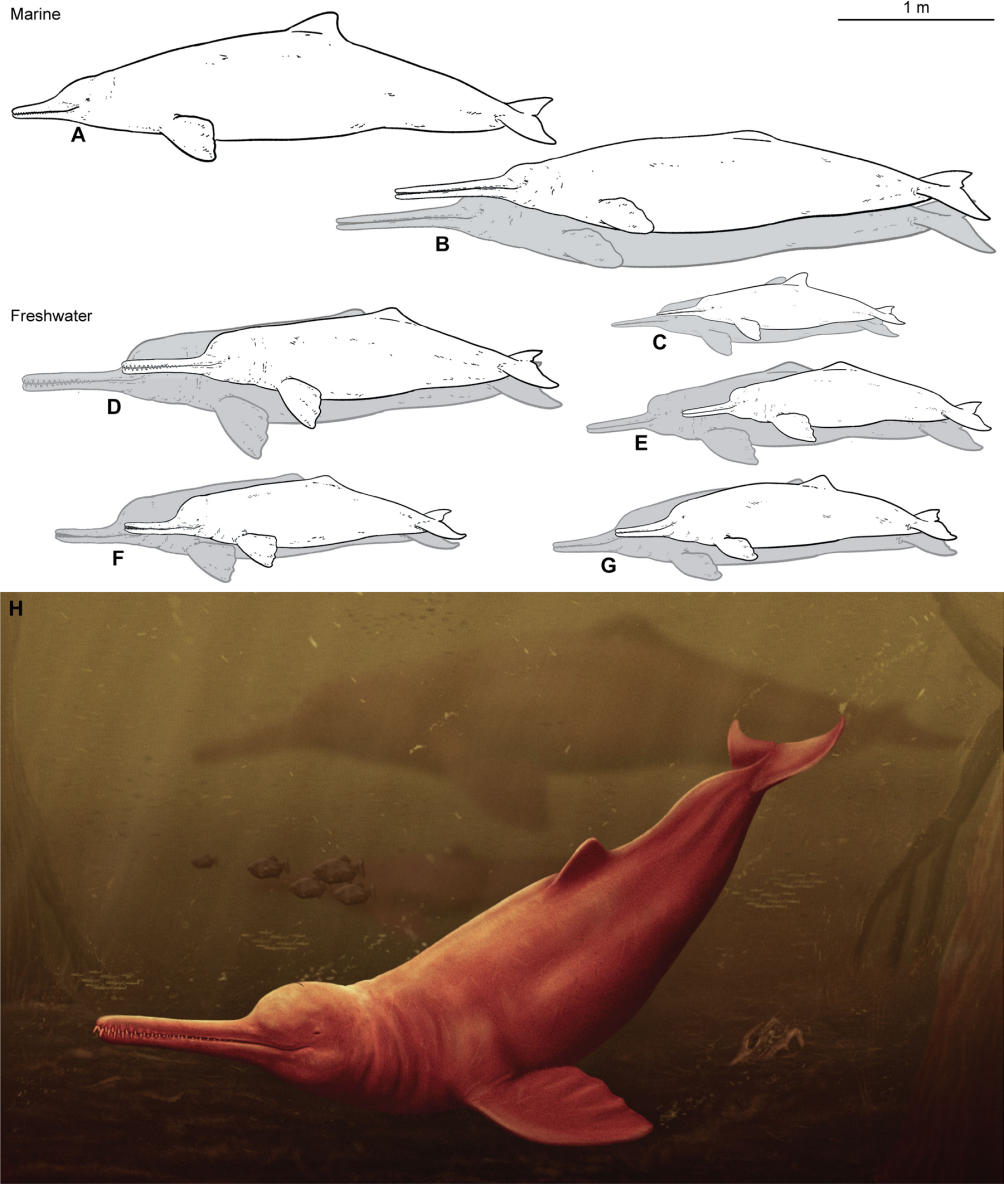
Size comparison between “river dolphins” and marine platanistoids and river dolphins. White silhouettes indicate the minimum body length calculated or recorded; gray body outlines indicate the largest size recorded or estimated in: *Macrosqualodephis ukupachai^†^* (**A**), *Zarhachis flagellator^†^* (**B**), *P. blainvillei* (**C**), *Pebanista yacuruna^†^* gen. et sp. nov. (**D**), *Inia geoffrensis* (**E**), *Platanista gangetica* (**F**), and *Lipotex vexillifer* (**G**). Artistic reconstruction of *Pebanista yacuruna* gen. et sp. nov. by Jaime Bran (**H**).

The occurrence of *Pebanista* in Early to Middle Miocene layers of the Pebas Fm. in Peru (*45*, *46*) not only confirms the presence of platanistids in the South American continent but also indicates that these animals reached body lengths similar to those of their marine relatives (i.e., larger than extant freshwater dolphins). During the Early to Middle Miocene (23 to 10 Ma), most of the modern west Amazon rainforest area (i.e., in Colombia, Peru, and Brazil) was covered by continental-scale fresh water to brackish water foreland system (the Pebas System) parallel to the Andes (*47*, *48*), with at least two large-scale events of marine influx from the Caribbean (*49*–*53*). This basin formed by by flexural subsidence in response to the Andean tectonic loading since the earliest Miocene (*48*, *54*). The massive proto-Amazonian Pebas wetland system was established during the Early Miocene and reached its maximum extent during the Middle Miocene Climatic Optimum (*46*, *49*, *51*), creating a complex arrangement of terrestrial and aquatic environments rich in nutrients and prey types (*55*). Extraordinary faunal diversity inhabited this region, including a wide array of fishes, turtles, crocodylians (caimans and gharials), and small to large mammals (e.g., marsupials, sloths, rodents, primates, and ungulates), among others (*15*, *56*–*58*). The diverse aquatic environment of the Pebas System, with widely varied and abundant food resources, might have greatly benefited the evolution of large predators, such as *Pebanista* and gharials, the latter a group of longirostrine crocodylians with extant representatives in southeast Asia. *Pebanista* and South American gharials, such as *Gryposuchus*, display an analogous evolutionary pattern, in which marine ancestors invaded and diversified in freshwater environments during the Neogene (*58*). Furthermore, abundance of similar prey items suitable for longirostrine forms and favourable environmental conditions might have prompted the evolution of gigantism among platanistids and *Gryposuchus* (*58*, *59*), markedly exceeding the size of their modern relatives (Fig. 4). The evolution of such large sizes in *Pebanista* and coeval crocodilians (*60*) could be related to a red-queen pattern of size increase, as a result of competitive interactions with other aquatic predators.

*Pebanista* is a new fossil taxon of platanistid dolphin from the Early to Middle Miocene (c. 16.5 Ma) Pebas Fm. in the Amazon Basin of Peru, the first freshwater representative of this clade in South America, characterized by an asymmetrical skull, large and robust rostrum, greatly developed supraorbital crests, and a circumnarial basin extending onto the whole facial region. *Pebanista* is recovered in our phylogenetic analyses as the closest known relative of extant *Platanista* from South Asia, sharing several synapomorphies with the latter, but also a combination of morphological characters that indicate a transitional stage between marine and freshwater habitats. The presence of this Early to Middle Miocene platanistid dolphin confirms the existence of this group in freshwater habitats of the Andean foreland basin system of the South American continent, long before the independent invasion of South American freshwater environments by the Amazon river dolphin lineage (Iniidae, *Inia*). After reaching the continental interior, *Pebanista* would have encountered the extraordinarily rich Pebas mega-wetland communities and vast freshwater environments of proto-Amazonia, thousands of kilometers, and oceans away from the range of extant *Platanista*, during a time in which the unusually broad diversity and great abundance of food resources also would have promoted its evolution toward a greater body size (Fig. 4).

## MATERIALS AND METHODS

### Phylogenetic analysis

To investigate the phylogenetic relationships of *Pebanista* within Platanistoidea, MUSM 4017 was coded in the morphological matrix of Bianucci *et al.* (*21*) using Mesquite 3.70 (*61*), resulting in a total of 24 operational taxonomic units and 48 morphological characters. Cladistic parsimony analyses were performed in PAUP 4.0a169 (*62*) via heuristic searches using the tree bisection-reconnection algorithm and treating all characters as unordered. Because of the high number of poorly supported nodes (less than 50% bootstrap support) and polytomies resulting from the first parsimony analysis, a series of analyses were performed by down-weighting of homoplastic characters with *k* values of 2, 3, 10, 20, and 40 (*22*, *23*). Because the topology remained unmodified, the lowest value was kept (*K* = 2). Both strict and Adams consensus trees were determined for each analysis, as was the statistical support for each, on the basis of 1000 bootstrap replicates.

### Body size

The body length of *Pebanista* was estimated on the basis of its bizygomatic width (BZW), using regression equations that reconstruct body size on the basis of this specific cranial measurement. However, prior studies (*21*) have suggested that body size reconstructions using the BZW underestimates the true body size in crown platanistoids. Therefore, the body size calculations presented in this study likely represent the minimum body sizes for this taxon. The BZW width of the specimen referred to cf. *Pebanista* MUSM 3593 was obtained using the proportion between the rostrum width at the base and the total BZW of the holotype specimen of *Pebanista yacuruna* MUSM 4017. Using this reconstructed BZW, the estimated body size of the referred specimen was calculated.

## Supplementary Materials

### This PDF file includes:

Supplementary Text Sections S1 to S3 Figs. S1 to S7 Table S1 References
