# Supplementary material for: The largest freshwater odontocete: A South Asian river dolphin relative from the proto-Amazonia
Source: Sci Adv. 2024 Mar 20;10(12):eadk6320. doi: 10.1126/sciadv.adk6320 (PMC11800881; doi:10.1126/sciadv.adk6320)
Supplement: Supplementary file 1 — Supplementary Text Sections S1 to S3 Figs. S1 to S7 Table S1 References [file sciadv.adk6320_sm.pdf]

Science Advances
AAAS

Supplementary Materials for

The largest freshwater odontocete: A South Asian river dolphin relative from the proto-Amazonia

Aldo Benites-Palomino *et al.*

Corresponding author: Gabriel Aguirre-Fernández, gabriel.aguirre@pim.uzh.ch

Sci. Adv. **10**, eadk6320 (2024)
DOI: 10.1126/sciadv.adk6320

This PDF file includes:

Supplementary Text
Sections S1 to S3
Figs. S1 to S7
Table S1
References

Supplementary Text

S1: Extended geological context

The holotype specimen of *Pebanista yacuruna* gen. et sp. nov., MUSM 4017, was uncovered from sediments of the Miocene Pebas Formation exposed along the Napo River in Western Amazonia of Peru (lat: -3.012468°, long: -73.404855°, Amazon Basin). The unit is associated with a large-scale continental freshwater aquatic system (fluvio-lacustrine setting), which was at least twice affected by short-lasting regional marine floodings sourced from Caribbean marine waters(*54*, *64*). The Pebas Fm. has an estimated thickness of up to 1000 meters(*46*). However, individual sections outcropping along riverbanks in the Amazon Basin rarely exceed a few tens of meters, making it virtually impossible to determine robust regional correlations and precise ages. The *Pebanista* locality consists of an 8-meter-thick section, characterized by having massive to finely laminated blue to brown siltstones, interbedded with brown fine-grained sandstones, occasional black fossiliferous mudstones, and a massive indurated fossiliferous coquina level. The *Pebanista* remains and other fossils found at the locality, such as mollusc shells, and bony remains of a *Purussaurus* caimanine and a sloth, come from this coquina level.

Palynological Biostratigraphy and Biochronological Implications–During our 2018 fieldwork campaign that yielded the holotype specimen of *Pebanista yacuruna*, we collected four samples (at 1-meter intervals) for palynological analyses from the section bearing the holotype. Two samples were located below (RN39-02; RN39-04) and two above (RN38-02; RN38-03) the stratigraphic position of the fossil specimen. Samples were processed for palynological analysis following standard digestion procedures by using 10% HCl and 40% HF(*65*). Each sample was sieved using 10 µm and 100 µm meshes and permanent montages were prepared. Light microscopy was used to examine the palynological content, following morphological features, descriptions, and illustrations compiled in Jaramillo and Rueda(*66*). The section was dated using the palynological zonation proposed by Jaramillo et al.(*19*), which has been time-calibrated using carbon isotopes, radioisotopic dating, foraminiferal biozonations, and magnetostratigraphic data. A Maximum Likelihood (ML) analysis was then applied to statistically infer the most probable ages based on taxa abundances(*67*), while keeping the same zonation as the biostratigraphic reference. R code and reference datasets were from Ochoa et al(*68*).

Good pollen recovery was found in all samples, being characterized by high abundances of fern spores (e.g., *Polypodiisporites* and *Laevigatosporites*) and palms (e.g., Mauritiidites). We found no evidence of taxa reflecting marine or brackish environments in this section. The co-occurrence of key temporal indicator taxa, such as *Malvacipolloides maristellae* (First Occurrence Datum or First Appearance Datum [FAD] at 17.7 Ma), *Echitricolporites spinosus* (FAD at 17.1 Ma), with *Cyclusphaera scabrata* (Last Occurrence Datum or Last Appearance Datum [LAD] at 16.9 Ma), and *Crassoretitriletes vanraadshooveni* (FAD at 14.2 Ma), supports the presence of the Early Miocene zones T-13, T-14 and the lower part of zone T-15 (17.7 -14.2 Ma) in this section. Furthermore, according to the ML analysis, the studied samples have a high probability of belonging to the Early Miocene palynological zone T-13 *E. maristellae*, restricting the age of the *Pebanista* section to the upper Burdigalian Stage, circa 16.5 Ma (Supplementary Figure 2).

S2: Extended description of *Pebanista yacuruna* gen. et sp. nov., MUSM 4017

Phylogenetic position of *Pebanista*–Our series of phylogenetic have consistently recovered the relationships of *Pebanista yacuruna* within Platanistidae. In the first heuristic search, 72 trees were retained with a length of 105 step, a CI=0.600 and a RI of 0.817. *Pebanista* + *Platanista* were recovered as sister to *Zarhachis* + *Pomatodelphis*; however, *Araeodelphis* was recovered within a large polytomy that included other platanistoids. In the series of analyses with implied weighting the content of Platanistidae was consistent with previous workers (*21*). In these analyses with subsequent k values, 13 trees were retained with a length of 13 steps, a CI=0.600 and a RU=0.817. Platanistidae was recovered by the following combination of characters: articular rim on the lateral surface of the periotic forming a hook-like process {c. 20(2)}, separate ossicle at the apex of the anterior process of the periotic {c. 26(1)}, outer posterior prominence of the tympanic posteriorly longer than the inner posterior prominence {c. 29(1)} and mandibular symphysis reaching more than 65% of the mandibular length, including an angle between both mandibles broader than 50° {c. 47(1)}. *Araeodelphis* is recovered as the earliest branching platanistid due to a single character: absence of asymmetry in the rostrum {c. 4(0)}. The clade formed by the more crownward platanistids, without *Araeodelphis* is recovered by apex of the rostrum formed by both premaxillae and maxillae {c. 2(2)}, deeply grooved lateral rostral suture between the premaxilla and the maxilla {c. 3(1)}, presence of a distinct prominent dorsal crest in the antorbital-supraorbital region {c. 9(1)}, antorbital process of the frontal transversely thickened {c. 10(1)}, lack of accessory denticles on posterior teeth {c. 33(1)}. The monophyly of *Pebanista* and *Platanista* is recognised due to large and thin-edged aperture of the cochlear aqueduct of the periotic {c. 22(1)}, oval internal auditory meatus of the periotic with the dorsal opening for the facial canal lateral to the spiral cribriform tract {c. 25(1)}, tooth count between 25 and 33 {c. 34(1)}, vertex strongly pinched transversely with the posterior end of the maxillae converging posterior to the external bony nares {c. 41(1)}, transversely wide temporal fossa {c. 44(0)}. *Pebanista* differs from *Platanista* by possessing a transversely thickened antorbital process of the frontal, ventral exposition of the palatines and greatly enlarged alveoli {c. 46 (0)}.

General description of the skull–The holotype skull of *Pebanista yacuruna* gen. et sp. nov., MUSM 4017, has a preserved condylobasal length of 698 mm and an estimated bizygomatic width of 281 mm. The preserved skull dimensions are proportionally larger than other Platanistidae including extant *Platanista*, and the fossil taxa Araeodelphis, *Pomatodelphis, Prepomatodelphis* and *Zarhachis*. The sutures between the cranial bones are well-closed or fused (e.g., maxilla-premaxilla suture along the rostrum) indicating that it belonged to a fully mature specimen. The rostrum is dorsoventrally flattened and elongated, a condition shared with *Pomatodelphis, Prepomatodelphis* and *Zarhachis*, thus contrasting with the transversely compressed rostrum of *Platanista* (*38*, *39*). On the preserved portion, the rostrum is formed by the premaxillae, maxillae, and vomer, being much more transversely robust than in other platanistids. The rostrum exhibits several well-preserved dental alveoli which are well-developed, much larger, and deeper than in other platanistids. The facial region of the skull exhibits a well-developed circumnarial basin, delimited laterally by the supraorbital crest, and posteriorly by the nuchal crest. The external bony nares are displaced to the left, creating an asymmetric arrangement of the surrounding bones. Only the left supraorbital crest is preserved, projecting substantially over the level of the base of the

rostrum and being transversely flattened as in *Platanista*, but more robust. This crest is not as stout as in *Pomatodelphis* and *Zarhachis*. The medial aspect of the crest is excavated by various vacuities, a condition also present in *Platanista*, but to a lesser extent in the new fossil taxon. No excavation of the supraorbital crest is observed in *Zarhachis* and *Pomatodelphis*. The temporal fossa is transversely longer than high and extends posteriorly, projecting the temporal crest well into the occipital region. Over the temporal fossa, the nuchal crest continues anteriorly, forming supraorbital crests and giving the skull a boxy outline in dorsal view. The occipital shield is perpendicular to the longitudinal axis of the skull, except for its dorsal end, which deflects anteriorly. However, it is not possible to assess whether this is the true shape of the crest, or a condition enhanced by taphonomic compression.

Premaxilla–Along the rostrum, the dorsal surface of the premaxillae is flat to slightly convex until the level of the antorbital notch, where the medial part of these deepens forming the circumnarial basin. Posteriorly, across the facial region of the skull, the premaxillae become slightly concave medially forming the premaxillary sac fossae. In dorsal view, the premaxillae are greatly exposed along the rostrum, limiting the dorsal exposure of the maxillae to a narrow strip along most of the rostrum. Both premaxillae retain a similar transverse width along the rostrum, without any narrowing or lateral expansion. The maxillary-premaxillary suture is well-closed and partly fused, being located within a narrow, shallow groove, much thinner than the condition observed in other platanistids such as *Pomatodelphis*, *Zarhachis* or *Platanista,* which exhibit a deep lateral groove. The mesorostral groove is closed along most of the rostrum, opening posteriorly at the level of the last maxillary tooth. The anteromedial sulcus is well-developed on the left premaxilla, being narrower on the right premaxilla due to the transverse compression of the bone, a condition shared with other platanistids and squalodephinids. The single premaxillary foramen is located on the left premaxilla, 21mm anterior to the level of the external bony nares, being longer (15mm) than wide (6mm). As in *Platanista*, this single premaxillary foramen is located at the level of the antorbital notch, and not anterior to it. The posteromedial sulcus is slightly narrower than its anteromedial counterpart, being mostly rectilinear on the left premaxilla and slightly curved on the right. On the facial region both premaxillae are strongly deflected to the left side of the skull, thus making the left external bony naris longer than wide and the right naris wider than long. Both premaxillae reach their highest elevation posterior to the external bony nares at the level of their respective nasal processes. The premaxillary sac fossa is well-developed on the left premaxilla, having a kidney-like shape. Because of the transverse compression, the premaxillary sac fossa on the right exhibits an oval outline.

Maxilla–The maxillae are mostly restricted to the lateral sides of the rostrum, being flat across the rostral surface. Anterior to the rostral base, the maxillae slightly project laterally and flatten, creating a blade-like ending of the maxillary flange, as in other platanistids. Ventrally, the maxillary surface is flat, and both maxillae contact each other until the level of the 13th dental alveolus (counting from the back), where the vomer is exposed ventrally as a narrow strip. The widest alveolus has an anteroposterior length of 16mm and a transverse width of 18mm. Posterior to the maxillary flange, the antorbital notch displays an acute V-shaped outline. A single dorsal infraorbital foramen is preserved on the left maxilla, displaying a sub-elliptical outline, reaching an anterodorsal length of 11mm and a transverse width of 4mm. No additional foramina can be identified within the maxillary surface. In the facial region, both maxillae are highly asymmetric, due to the deviation of the external bony nares, following the pattern of their respective premaxilla.

The lateral region of the ascending process of the maxilla projects dorsolaterally, forming the U-shaped circumnarial basin along with the premaxillae. Such development of the circumnarial basin is a condition unique to *Pebanista*, being a more-derived condition than the one observed in *Platanista*, and because of the overall contribution of the premaxillae and maxillae to the structure it resembles the supracranial basin observed in other odontocetes(*32*, *33*, *69*). Laterally, the circumnarial basin is surrounded by the greatly enlarged supraorbital crests, which are mostly formed by the frontals. The maxillae only contribute to a minor part of the medial wall of the supraorbital crests, at the level of the temporal fossa. Posteriorly, and within the circumnarial basin there are two posterolateral depressions located on the floor of the basin, each of which display a somewhat symmetrical outline.

Frontal + lacrimal–Most of the left frontal is preserved, except for the dorsal edge of the supraorbital crest. The right frontal has been lost during the taphonomic process, except for a small bony strip. The suture between the maxilla and the frontal is mostly fused until the level of the temporal fossa, where the suture between both bones can be identified posteriorly. Because of the shape and the overall development of the circumnarial basin, both frontals should have reached a similar height and anterodorsal projection, with minor differences due to the asymmetric profile of the facial region. In *Pebanista*, the frontals are more enlarged than the condition observed in other platanistoids, due to the great dorsal development of the supraorbital crests. The frontal is exposed lateral to the maxillae in dorsal view, displaying a transversely robust profile. In the circumnarial basin, the frontal greatly elevates over the rest of the facial region, forming the supraorbital crest, which is transversely thinner than in *Zarhachis* and *Pomatodelphis*, although not as thin as in *Platanista*. Furthermore, the dorsomedial region of the crest is excavated by several large vacuities, which may foreshadow the condition of *Platanista*, in which the medial aspect of the crest is totally excavated to house a dorsal expansion of the pterygoid sinus (*70*). In dorsal view, the lateral edge of the orbit roof is not parallel to the anteroposterior axis of the skull as in *Dilophodephis*, but forms an acute angle with the rostrum base, being slightly medially oriented. In other words, the orbits face anterolaterally. Ventrally, only a small portion of the lacrimal can be recognised anteriorly contributing to the preorbital process. The frontal groove is moderately broad and shallow, indicating that optic nerve development was limited as compared to *Zarhachis* or *Pomatodelphis*, thus indicating an intermediate condition between these two genera and the nearly blind *Platanista*. Such condition is also reflected by the proportional reduction in the size of the orbit.

Palatine + pterygoid –The palatine and pterygoid are not easy to distinguish, as the palatal in the holotype specimen is compressed and broken into several small bony fragments, and the palatine-pterygoid suture is fused. In *Pebanista*, the pterygoids have an anterior projection with a round outline. Medially, the pterygoids contact each other but are not fused. Lateral to pterygoid, the palatine is ventrally exposed as a small stripe of bone. Most of the medial part of the palatine has been covered by the pterygoid. As in several platanistoids, the palatine projects dorsolaterally. Both pterygoid sinus fossae are well-developed, displaying an elongated bean-like shape, extending from the level of the antorbital notch to the level of the anterior tip of the squamosal. The hamular process of the pterygoid is mostly missing except for a small area anterior to the choanae. Posteriorly the pterygoid extends towards the pharyngeal and basioccipital crests, contributing to most of their preserved length.

Temporal Region–The skull has undergone a slight to moderate dorsoventral compression during the taphonomic process, being more evident on the right side of the skull, as the temporal crest suffers an abrupt break on its posteriormost portion. Despite this, the overall shape of the temporal fossa has been mostly preserved, presenting an oval outline in lateral view, being transversely broad and much longer anterodorsally than high. The overall extension of the temporal fossa in *Pebanista* is much greater than that of *Platanista* or *Zarhachis*. The fossa projects into the occipital region, displacing the temporal crest to the occipital region, resembling the condition in *Zarhachis*.

Squamosal–Both squamosals are partly preserved, each to a similar extent. Only part of the left zygomatic process is preserved, being much more robust than in *Platanista*, but more slender than in *Zarhachis* or *Pomatodelphis*. The supramastoid crest is only noticeable in dorsal view, near the posterior end of the bone, being broad but not very high. Despite this, the process should have been moderately robust, based on the broken surface of the squamosal. The glenoid fossa is small, with an elliptical outline, and oriented towards the anterolateral portion of the skull.

Occipital Region–*Pebanista* displays an occipital region with a trapezoidal outline delimited by the nuchal crest and the temporal crests. The surface of the occipital shield is mostly perpendicular to the skull main axis, except for its dorsal part, which projects anteriorly. The supraoccipital and the posterior end of the maxilla contribute to the anterodorsally oriented nuchal crest. The foramen magnum and the occipital condyles have been dorsoventrally compressed; despite this, the overall shape of the surrounding bones indicates that the foramen should have had a mostly circular outline. The occipital condyles protrude markedly posteriorly, having a rounded to triangular shape in posterior view.

S3: Description of additional referred specimens

cf. *Pebanista*

Referred specimen– MUSM 3593, an isolated rostral fragment.

Locality, Age and Horizon– This specimen was donated by Juan Pablo de Molina in 2018, and was recovered from the Fitzcarrald Arch, an area in South Central Peru where the middle Miocene Ipururo Fm. crops out. Despite not having a well-defined locality, the sediments attached to the rostral fragment indicate that it corresponds to the fluvial or tidal deposits in this area. Previous work in the area indicates that the Fitzcarrald Arch was a highly diverse region during the middle Miocene, as a wide array of taxa including, but not restricted to, crocodilians, cingulates, folivores, astrapotheres, notoungulates, litopterns, rodents, odontocetes and a possible marsupial have been reported so far(*57*, *71*).

Description– The rostral fragment is rather robust and reaches a maximum length of 13.5 cm and a maximum width of 8.8 cm, including the premaxillae, maxillae, and vomer. In dorsal view, the maxilla is restricted to a narrow strip lateral to its respective premaxilla. Medially the suture between both premaxillae is somewhat noticeable, being slightly deviated to the left side. The premaxilla-maxilla suture is located within a narrow, moderately shallow recess, like the condition observed in the holotype skull of *Pebanista*. The lateral walls of the rostrum are parallel, except for a minor distortion resulting from to the taphonomic process. Ventrally, both maxillae preserve four well-developed dental alveoli on each side, with the broken teeth preserved in situ within these alveoli. The posteriormost right alveolus reaches an anterodorsal length of 2.9 cm and a transverse width of 1.9 cm. Only two greatly enlarged teeth are preserved on the left maxilla. The largest one reaches a 2.4 cm exposure of the crown and displays several longitudinal ornamentations on the crown. The tooth resembles that of *Platanista*, but proportionally much larger in this fossil. Medially, the vomer is exposed as a narrow strip between both maxillae.

Platanistidae indet.

Referred specimen– MUSM 4759, isolated tympanic.

Locality, Age and Horizon– The isolated tympanic was found in coquina layers of the lower Pebas Fm. (Early to Middle Miocene) along the Napo River.

Description– The isolated tympanic reaches a maximum length of 5.9 cm, being anteroposterioly longer than wide, and resembles in general shape that of other platanistoids such as *Platanista, Pomatodelphis* or *Notocetus* (*40*, *44*). In MUSM 4759 the anterior spine is long, but its overall extension cannot be assessed, as the distal tip is missing. Only the base of the anterodorsal crest is preserved, as the tympanic plate has only been partly preserved. The involucrum is well-developed, being rather robust due to the pachyosteosclerotic growth of the bone. The outer lip exhibits a minor extension anteriorly, similar to the condition observed in *Zarhachis* or *Platanista*. The preserved portion of the outer lip displays a moderately inflated outline, interrupted by the thin and shallow lateral furrow. Posteriorly, the outer posterior prominence has a moderately triangular profile in ventral view, contrasting with the inner posterior prominence, which displays a much rounder profile. The interprominential notch is wide and moderately shallow, along with the median furrow which extends across most of the ventral surface of the tympanic.

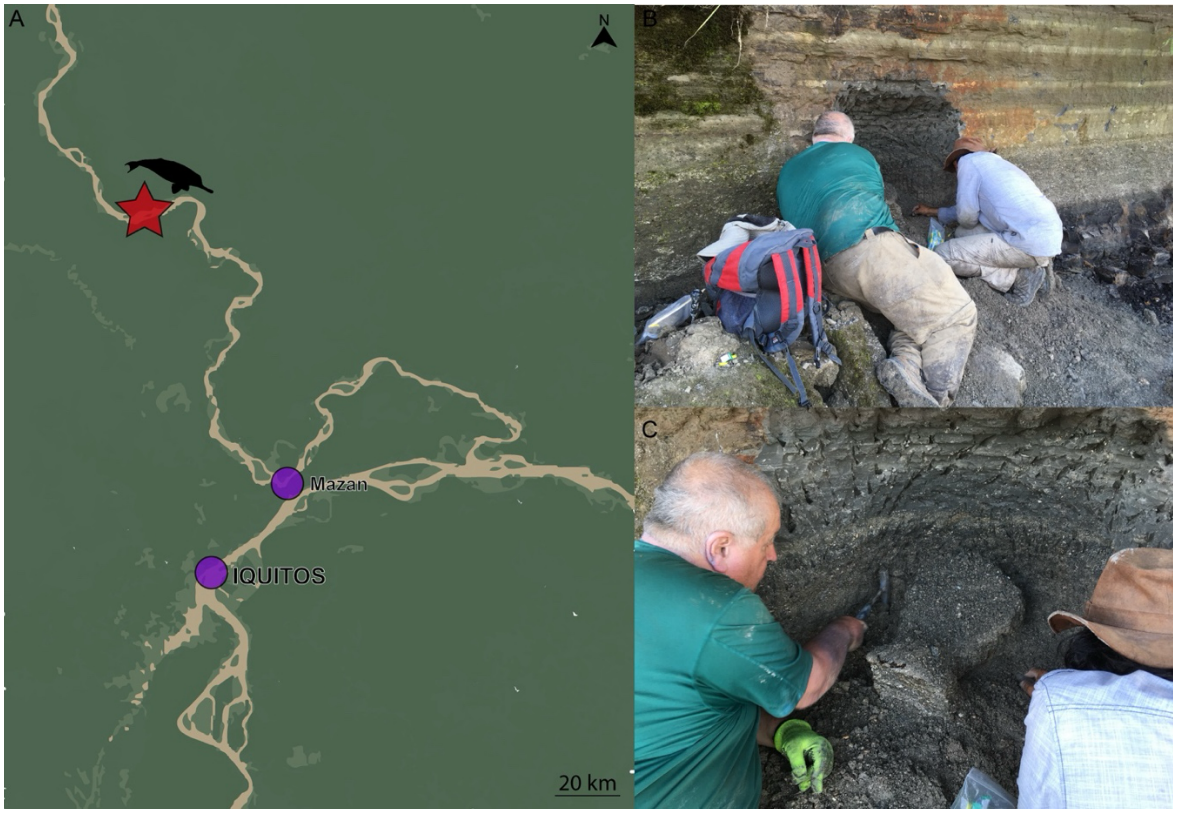

Fig. S1. Geographical context. Map indicating the locality where the holotype specimen of *Pebanista yacuruna* gen. et sp. nov., MUSM 4017, was found (A) and photos from the collection of the specimen (B, C) along the banks of the Napo River.

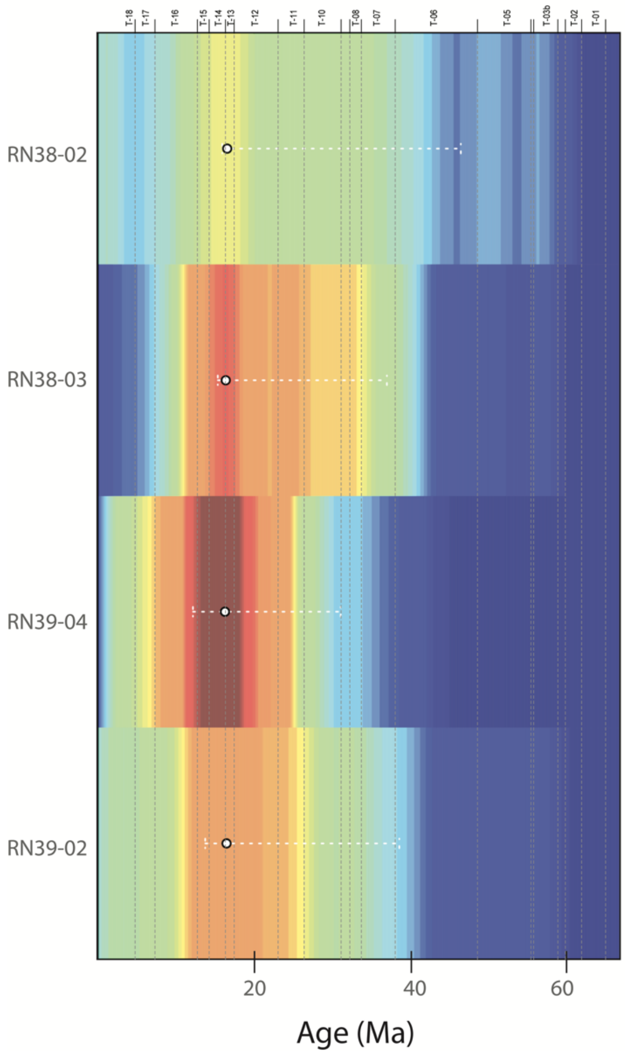

Fig. S2. Stratigraphic ML age estimates for the MUSM 4017-bearing section, calculated using a probabilistic approach based on the taxa abundances. Normalized likelihood values are represented by color, with higher and lower likelihood values symbolized by warmer and colder colors, respectively. The lower horizontal axis shows geologic time (Ma), and vertical axis represents stratigraphic position of specific samples and the upper horizontal axis marks the palynological zones proposed by Jaramillo et al.(*19*).

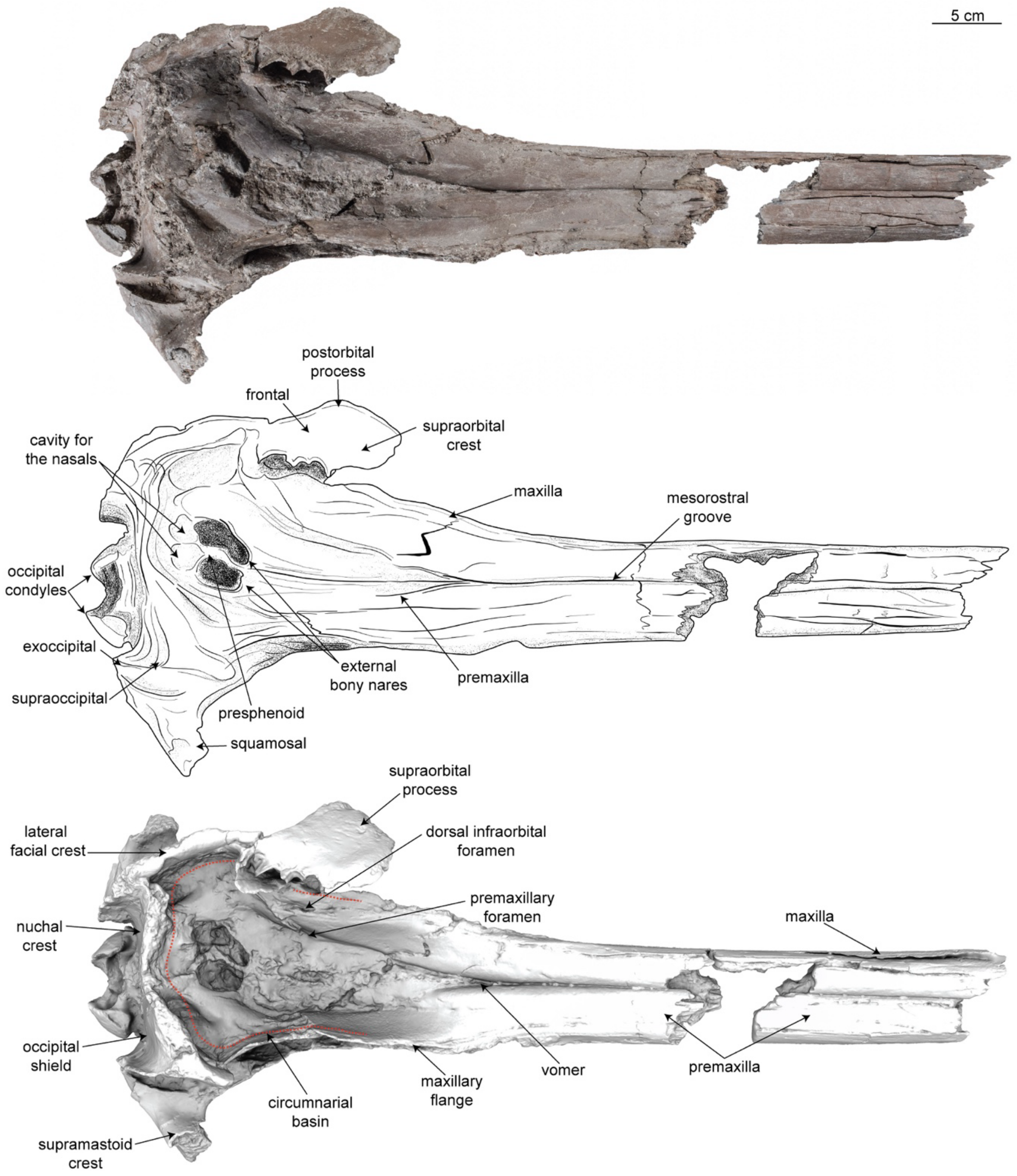

Fig. S3. Holotype skull of *Pebanista yacuruna* gen. et sp. nov. (MUSM 4017). Photograph (top), drawing (middle), and surface (bottom) 3D model in dorsal view.

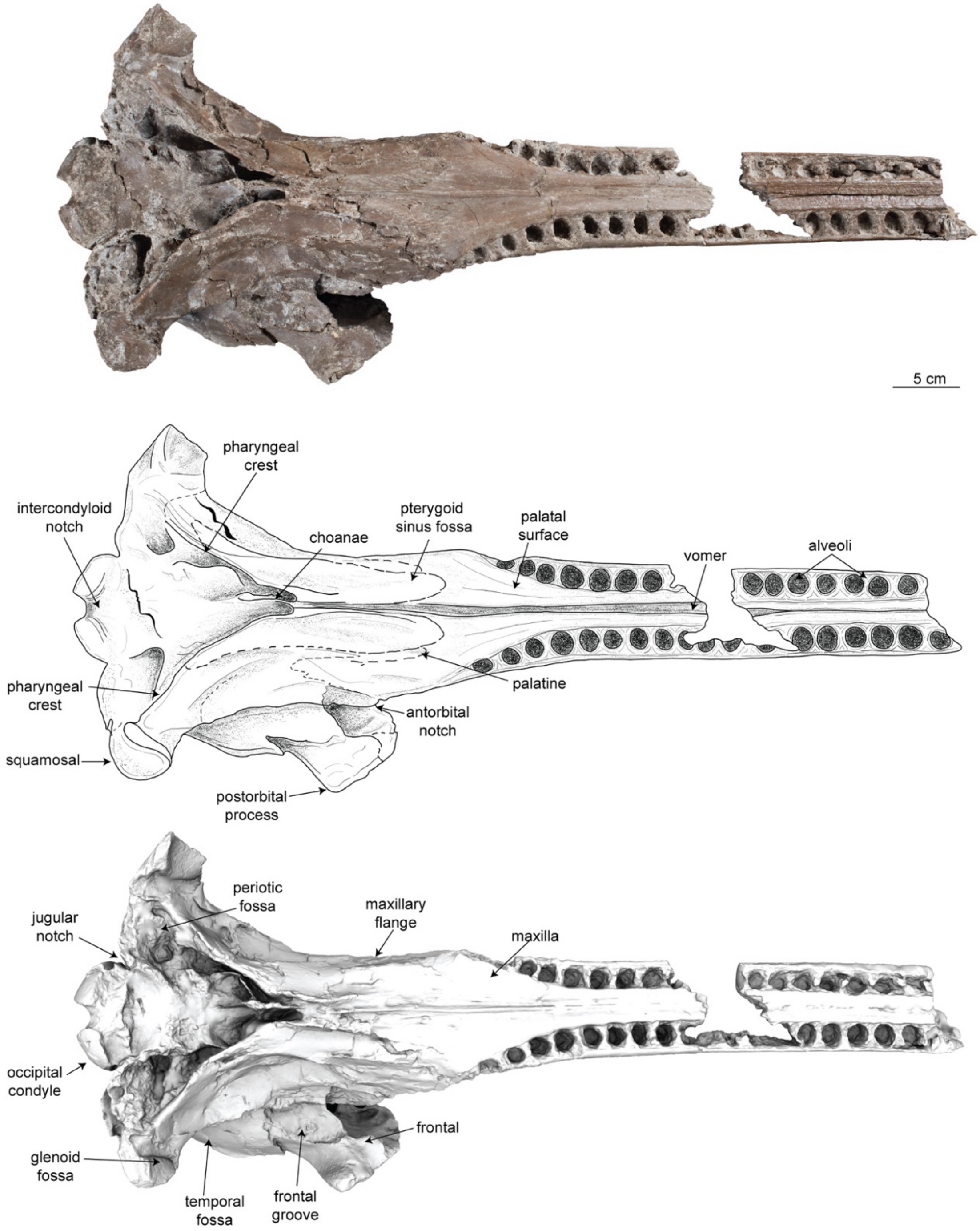

Fig. S4. Holotype skull of *Pebanista yacuruna* gen. et sp. nov. (MUSM 4017). Photograph (top), drawing (middle), and surface (bottom) 3D model in ventral view.

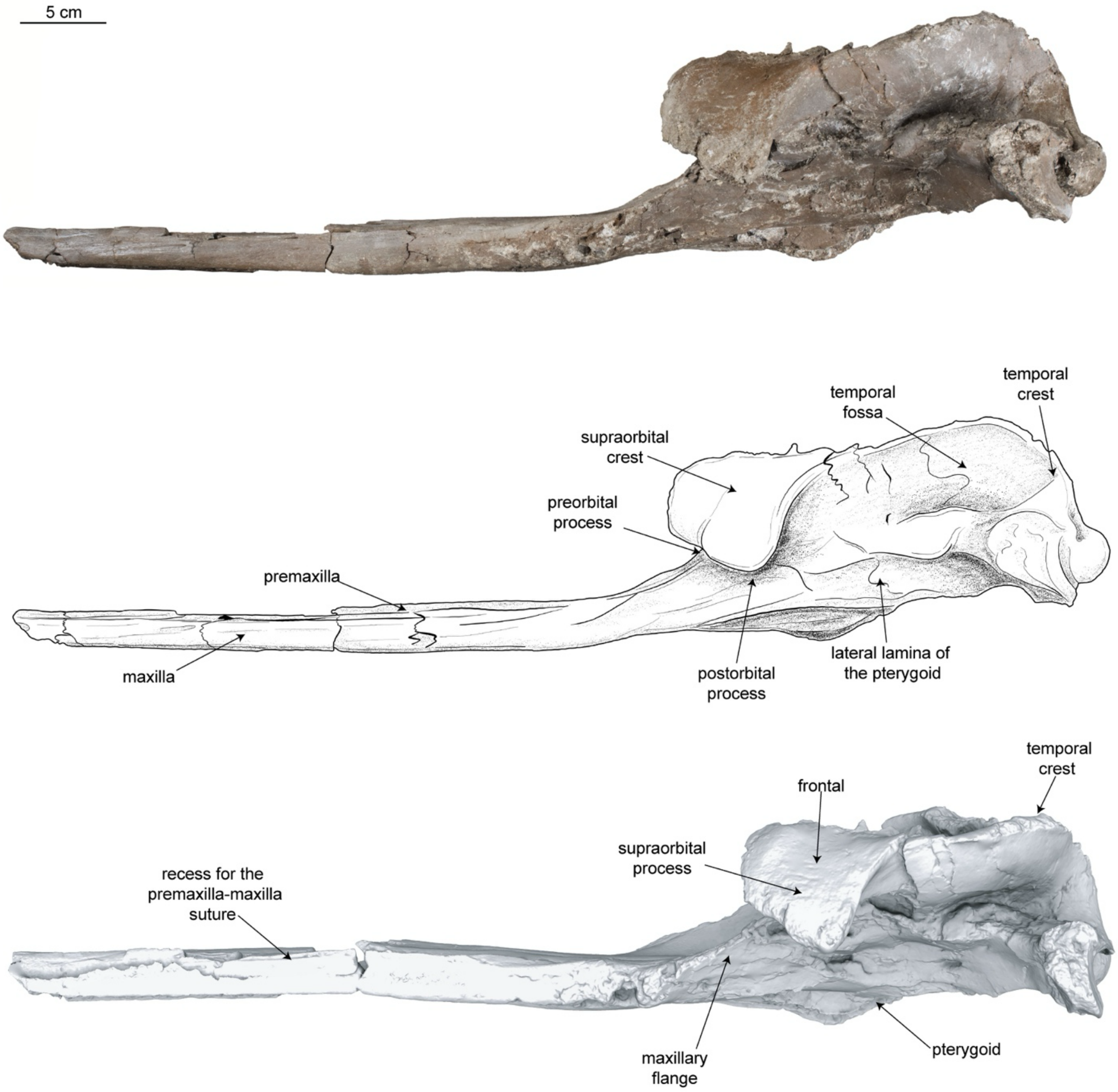

Fig. S5. Holotype skull of *Pebanista yacuruna* gen. et sp. nov. (MUSM 4017). Photograph (top), drawing (middle), and surface (bottom) 3D model in left lateral view.

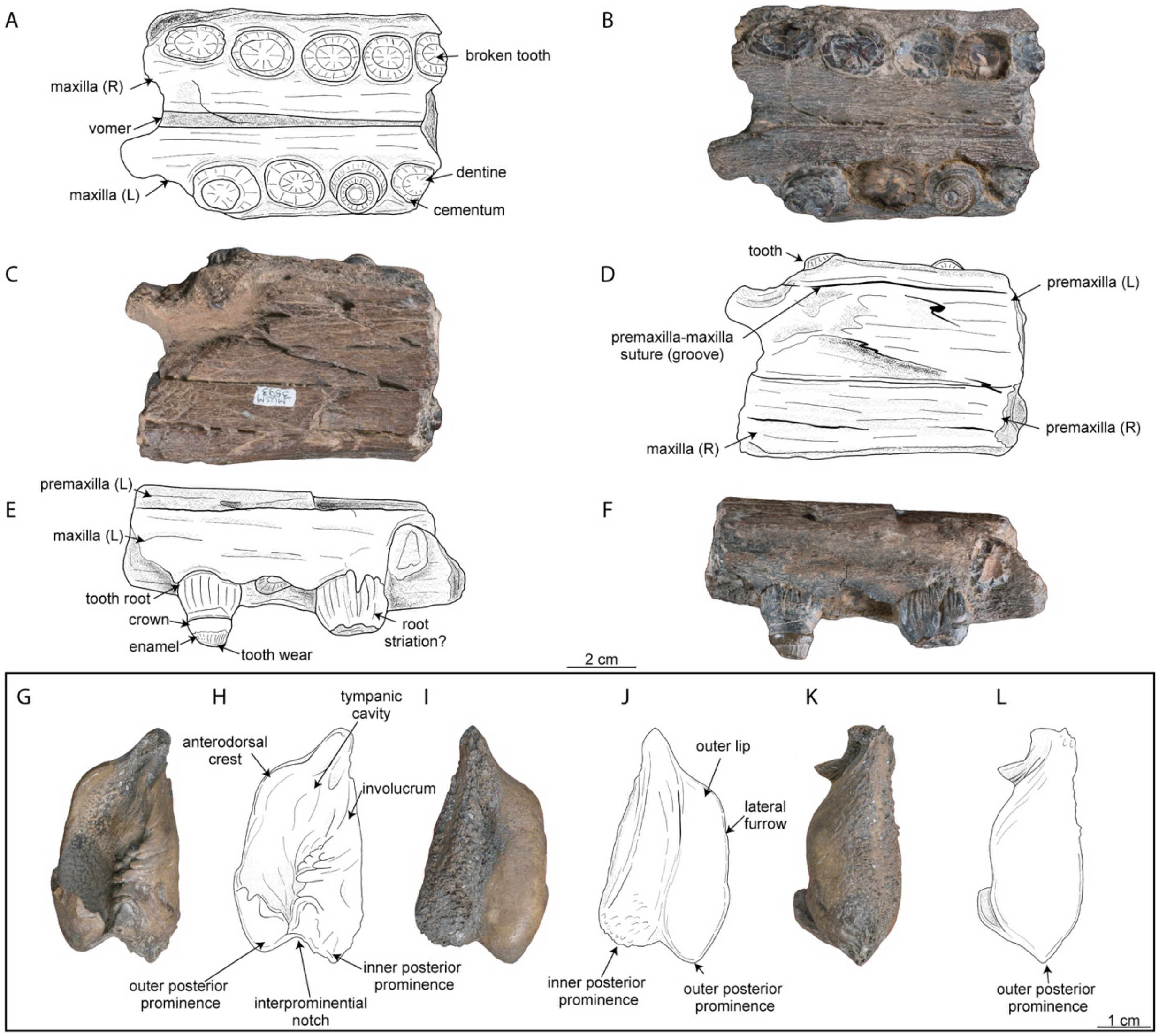

Fig. S6. Additional specimens: cf. *Pebanista* MUSM 3593 rostral fragment in ventral (A,B), dorsal (C,D) and right lateral (E, F) views; Platanistidae indet. MUSM 4017 isolated tympanic in dorsal (G,H), ventral (I, J) and lateral (K, L) views.

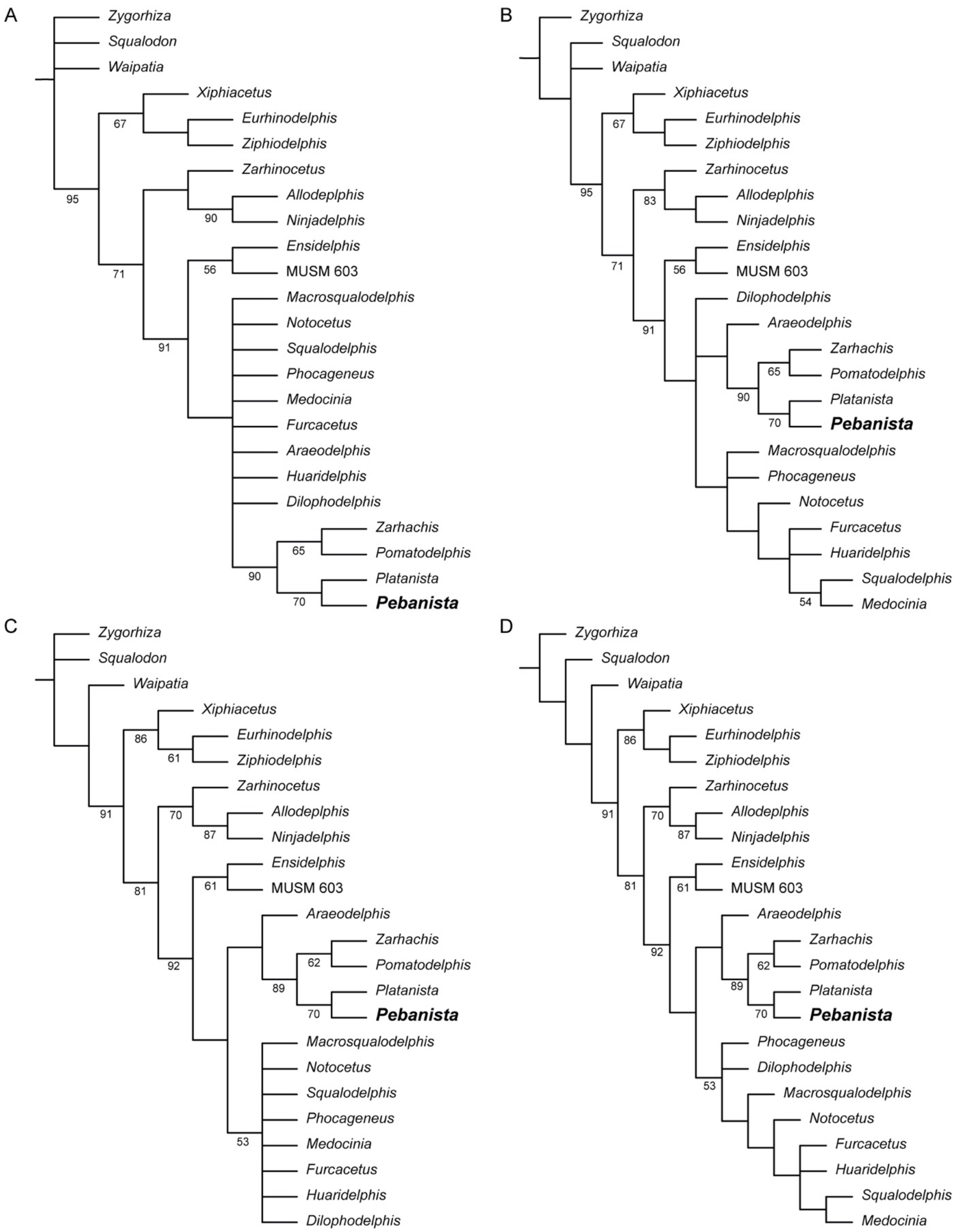

Fig. S7. Phylogenetic relationships of *Pebanista yacuruna* gen. et sp. nov. (MUSM 4017). Related to Figure 2. Strict (a) and Adams (b) consensus trees of the heuristic search without downweighting of characters; and with down-weighting (c,d) homoplastic characters (k=2), indicating the relationships of *Pebanista yacuruna* gen. et sp. nov. (MUSM 4017) to other platanistoids.

Table S1. Cranial measurements (e, estimate; + measurement on incomplete element) of the holotype skull of *Pebanista yacuruna* gen. et sp. nov., MUSM 4017 (in mm)

**Dimensions**	**MUSM 4017**
Condylobasal length (preserved)	698+
Length of the rostrum (preserved)	489+
Width of rostrum at base	116
Width of the rostrum at 60mm anterior to the line across hindmost limits of antorbital notches	102
Width of rostrum at mid preserved length	66
Width of premaxillae at mid preserved length of the rostrum	71
Width of rostrum at 3⁄4 of the preserved length, measured from posterior end (taken on preserved portion)	74
Height of rostrum at base (including rostral basin)	46
Height of rostrum at base (without rostral basin)	27
Height of rostrum at mid preserved length	21
Maximum width of the external right bony naris	22
Maximum width of the external left bony naris	18
Greatest postorbital width (width across postorbital processes)	261
Minor width within the supraorbital processes	115
Maximum width of external nares	46
Greatest width across zygomatic processes of squamosal	281e
Greatest width of premaxillae	94
Width across both the temporal fossae (minimum)	131
Greatest width between lateral margins of exoccipitals	242e
Dorsoventral length of temporal fossa	94
Anteroposterior width of temporal fossa perpendicular to greatest length (dorsoventral height)	124
Length of left orbit from apex of preorbital process of frontal to apex of postorbital process	54+
Length of antorbital process of left lacrimal taken horizontally	17e
Greatest width of internal nares	23
Greatest length of pterygoid	134
Maximum width across occipital condyles	78
Height of foramen magnum	21e
Width of foramen magnum	35

REFERENCES AND NOTES

1. J. H. Geisler, M. R. McGowen, G. Yang, J. Gatesy, A supermatrix analysis of genomic, morphological, and paleontological data from crown Cetacea. *BMC Evol. Biol.* **11**, 112 (2011).

2. C. de Muizon, O. Lambert, G. Bianucci, "River dolphins, evolution" in *Encyclopedia of Marine Mammals*, B. Würsig, J. G. M. Thewissen, K. M. Kovacs, Eds. (Academic Press, ed. 3, 2018), pp. 829–835; www.sciencedirect.com/science/article/pii/B9780128043271002211.

3. H. Hamilton, S. Caballero, A. G. Collins, R. L. Brownell Jr, Evolution of river dolphins. *Proc. R. Soc. London Ser. B Biol. Sc.* **268**, 549–556 (2001).

4. N. D. Pyenson, Requiem forLipotes: An evolutionary perspective on marine mammal extinction. *Mar. Mamm. Sci.* **25**, 714–724 (2009).

5. S. T. Turvey, R. L. Pitman, B. L. Taylor, J. Barlow, T. Akamatsu, L. A. Barrett, X. Zhao, R. R. Reeves, B. S. Stewart, K. Wang, First human-caused extinction of a cetacean species? *Biol. Lett.* **3**, 537–540 (2007).

6. G. Veron, B. D. Patterson, R. Reeves, Global diversity of mammals (Mammalia) in freshwater. *Hydrobiologia* **595**, 607–617 (2008).

7. P. E. Purves, G. Pilleri, "Observations on the ear, nose, throat and eye of *Platanista indi*" in *Investigations on Cetacea*, G. Pilleri, Ed. (University of Bern, 1973), pp. 13–57.

8. G. T. Braulik, F. I. Archer, U. Khan, M. Imran, R. K. Sinha, T. A. Jefferson, C. Donovan, J. A. Graves, Taxonomic revision of the South Asian River dolphins (Platanista): Indus and Ganges River dolphins are separate species. *Mar. Mamm. Sci.* **37**, 1022–1059 (2021).

9. O. Lambert, G. Bianucci, M. Urbina, J. H. Geisler, A new inioid (Cetacea, Odontoceti, Delphinida) from the Miocene of Peru and the origin of modern dolphin and porpoise families. *Zool. J. Linn. Soc.* **179**, 919–946 (2017).

10. N. D. Pyenson, J. Vélez-Juarbe, C. S. Gutstein, H. Little, D. I. Vigil, A. O'Dea, Isthminia panamensis, a new fossil inioid (Mammalia, Cetacea) from the Chagres Formation of Panama and the evolution of 'river dolphins' in the Americas. *PeerJ* **3**, e1227 (2015).

11. O. Lambert, C. Auclair, C. Cauxeiro, M. Lopez, S. Adnet, A close relative of the Amazon river dolphin in marine deposits: A new Iniidae from the late Miocene of Angola. *PeerJ* **6**, e5556 (2018).

12. M. A. Cozzuol, "Mamiferos acuaticos del Mioceno medio y tardío de Argentina Sistemática, evolución y biogeografía," thesis, Universidad Nacional de La Plata (1993).

13. G. Bianucci, O. Lambert, R. Salas-Gismondi, J. Tejada, F. Pujos, M. Urbina, P. O. Antoine, A Miocene relative of the Ganges River dolphin (Odontoceti, Platanistidae) from the Amazonian Basin. *J. Vertebr. Paleontol.* **33**, 741–745 (2013).

14. A. Benites-Palomino, G. Aguirre-Fernández, J. W. J. W. Moreno-Bernal, A. Vanegas, C. Jaramillo, Miocene freshwater dolphins from La Venta, Huila, Colombia suggest independent invasions of riverine environments in Tropical South America. *J. Vertebr. Paleontol.* **40**, e1812078 (2020).

15. J. D. Carrillo-Briceño, O. A. Aguilera, A. Benites-Palomino, A. S. Hsiou, J. L. Birindelli, S. Adnet, E.-A. Cadena, T. M. Scheyer, A historical vertebrate collection from the Middle Miocene of the Peruvian Amazon. *Swiss J. Palaeontol.* **140**, 26 (2021).

16. W. W. Schwarzhans, O. A. Aguilera, T. M. Scheyer, J. D. Carrillo-Briceño, Fish otoliths from the middle Miocene Pebas Formation of the Peruvian Amazon. *Swiss J. Palaeontol.* **141**, 2(2022).

17. J. W. Moreno-Bernal, Size and paleoecology of giant Miocene South American crocodiles (Archosauria: Crocodylia). *J. Vertebr. Paleontol.* **27**, 120A (2007).

18. E. A. Cadena, T. M. Scheyer, J. D. Carrillo-Briceño, R. Sánchez, O. A. Aguilera-Socorro, A. Vanegas, M. Pardo, D. M. Hansen, M. R. Sánchez-Villagra, The anatomy, paleobiology, and

evolutionary relationships of the largest extinct side-necked turtle. *Sci. Adv.* **6**, eaay4593 (2020).

19. C. A. Jaramillo, M. Rueda, V. Torres, A palynological zonation for the Cenozoic of the Llanos and Llanos Foothills of Colombia. *Palynology* **35**, 46–84 (2011).

20. A. T. Boersma, M. R. McCurry, N. D. Pyenson, A new fossil dolphin Dilophodelphis fordycei provides insight into the evolution of supraorbital crests in Platanistoidea (Mammalia, Cetacea). *R. Soc. Open Sci.* **4**, 170022 (2017).

21. G. Bianucci, C. de Muizon, M. Urbina, O. Lambert, Extensive diversity and disparity of the early miocene Platanistoids (Cetacea, Odontoceti) in the Southeastern Pacific (Chilcatay Formation, Peru). *Life* **10**, 27 (2020).

22. P. A. Goloboff, Extended implied weighting. *Cladistics* **30**, 260–272 (2014).

23. P. A. Goloboff, J. M. Carpenter, J. S. Arias, D. R. M. Esquivel, Weighting against homoplasy improves phylogenetic analysis of morphological data sets. *Cladistics* **24**, 758–773 (2008).

24. M. D. Nelson, M. D. Uhen, A new platanistoid, Perditicetus yaconensis gen. et sp. nov.(Cetacea, Odontoceti), from the Chattian–Aquitanian Nye Formation of Oregon. *J. Syst. Palaeontol.* **18**, 1497–1517 (2020).

25. M. Viglino, M. R. Buono, Y. Tanaka, J. I. Cuitiño, R. E. Fordyce, Unravelling the identity of the platanistoidNotocetus vanbenedeniMoreno, 1892 (Cetacea, Odontoceti) from the early Miocene of Patagonia (Argentina). *J. Syst. Palaeontol.* **20**, 2082890 (2022).

26. M. Roddaz, W. Hermoza, A. Mora, P. Baby, M. Parra, F. Christophoul, S. Brusset, N. Espurt, "Cenozoic sedimentary evolution of the Amazonian foreland basin system" in *Amazonia, Landscape and Species Evolution: A Look into the Past*, C. Hoorn, F.P. Wesselingh, Eds. (Wiley-Blackwell, 2010), p.61–88.

27. G. Bianucci, G. Bosio, E. Malinverno, C. D. Muizon, I. M. Villa, M. Urbina, O. Lambert, A new large squalodelphinid (Cetacea, Odontoceti) from Peru sheds light on the early miocene platanistoid disparity and ecology. *R. Soc. Open Sci.* **5**, 172302 (2018).

28. F. G. Marx, O. Lambert, M. D. Uhen, *Cetacean Paleobiology* (John Wiley & Sons, 2016).

29. A. Benites-Palomino, A. Reyes, G. Aguirre-Fernández, R. Sanchez, J. D. Carrillo-Briceño, M. Sanchez-Villagra, A stem Delphinidan from the Caribbean region of Venezuela. *Swiss J. Palaeontol.* **140**, 6 (2021).

30. C. M. Peredo, M. D. Uhen, M. D. Nelson, A new kentriodontid (Cetacea: Odontoceti) from the early Miocene Astoria Formation and a revision of the stem delphinidan family Kentriodontidae. *J. Vertebr. Paleontol.* **38**, e1411357 (2018).

31. G. Bianucci, C. D. Celma, M. Urbina, O. Lambert, New beaked whales from the late Miocene of Peru and evidence for convergent evolution in stem and crown Ziphiidae (Cetacea, Odontoceti). *PeerJ* **4**, e2479 (2016).

32. A. Benites-Palomino, J. Vélez-Juarbe, R. Salas-Gismondi, M. Urbina, Scaphokogia totajpe, sp. nov., a new bulky-faced pygmy sperm whale (Kogiidae) from the late Miocene of Peru. *J. Vertebr. Paleontol.* **39**, e1728538 (2020).

33. A. Benites-Palomino, J. Vélez-Juarbe, A. Collareta, D. Ochoa, A. Altamirano, M. Carré, M. J. Laime, M. Urbina, R. Salas-Gismondi, Nasal compartmentalization in Kogiidae (Cetacea, Physeteroidea): Insights from a new late Miocene dwarf sperm whale from the Pisco Formation. *Pap. Palaeontol.* **7**, 1507–1524 (2021).

34. O. Lambert, G. Bianucci, C. de Muizon, Macroraptorial sperm whales (cetacea, odontoceti, physeteroidea) from the miocene of peru. *Zool. J. Linn. Soc.* **179**, 404–474 (2017).

35. R. E. Fordyce, C. de Muizon, "Evolutionary history of cetaceans: A review" in *Secondary Adaptation of Tetrapods to Life in Water*, J.-M. Mazin, V. de Buffrénil, Eds. (Verlag Dr. Friedrich Pfeil, 2001), pp. 169–233.

36. S. J. Godfrey, L. G. Barnes, O. Lambert, The Early Miocene odontoceteAraeodelphis NatatorKellogg, 1957 (Cetacea; Platanistidae), from the Calvert Formation of Maryland, U.S.A, *J. Vertebr. Paleontol.* **37**, e1278607 (2017).

37. G. Aguirre-Fernández, J. D. Carrillo-Briceño, R. Sánchez, E. Amson, M. R. Sánchez-Villagra, Fossil cetaceans (Mammalia, Cetacea) from the neogene of Colombia and Venezuela. *J. Mamm. Evol.* **24**, 71–90 (2017).

38. L. G. Barnes, A phylogenetic analysis of the superfamily Platanistoidea (Mammalia, Cetacea, Odontoceti). *Beiträge zur Paläontologie* **30**, 25–42 (2006).

39. L. G. Barnes, An Early Miocene long-snouted marine platanistid dolphin (Mammalia, Cetacea, Odontoceti) from the Korneuburg Basin (Austria). *Beiträge zur Paläontologie* **27**, 407–418 (2002).

40. C. D. Muizon, The affinities of Notocetus vanbenedeni, an Early Miocene Platanistoid (Cetacea, Mammalia) from Patagonia Southern Argentina. *Am. Mus. Novit.* **2904**, 1–27 (1987).

41. D. P. Hocking, F. G. Marx, T. Park, E. M. G. Fitzgerald, A. R. Evans, A behavioural framework for the evolution of feeding in predatory aquatic mammals. *Proc. Biol. Sci.* **284**, 20162750 (2017).

42. G. Aguirre-Fernández, B. Mennecart, M. R. Sánchez-Villagra, R. Sánchez, L. Costeur, A dolphin fossil ear bone from the northern Neotropics–insights into habitat transitions in iniid evolution. *J. Vertebr. Paleontol.* **37**, e1315817 (2017).

43. C. S. Gutstein, M. A. Cozzuol, N. D. Pyenson, The Antiquity of Riverine Adaptations in Iniidae (Cetacea, Odontoceti) Documented by a Humerus from the Late Miocene of the Ituzaingó Formation, Argentina, *Anat. Rec.* **297**, 1096–1102 (2014).

44. T. Kasuya, "Systematic consideration of recent toothed whales based on the morphology of the tympano-periotic bone" in *The Scientific Reports of the Whales Research Institute*, T. Kasuya, Ed. [The Whales Research Institute (Tokyo, Japan), 1973], pp. 1–103.

45. F. P. Wesselingh, *Molluscan Radiations and Landscape Evolution in Miocene Amazonia* (2008).

46. F. P. Wesselingh, M. C. Hoorn, J. Guerrero, M. E. Räsänen, L. R. Pittman, J. Salo, The stratigraphy and regional structure of Miocene deposits in western Amazonia (Peru, Colombia and Brazil), with implications for late Neogene landscape evolution. *Scr. Geol.* 291–322 (2006).

47. F. P. Wesselingh, C. Hoorn, "Geological development of Amazon and Orinoco basins" in *Historical Biogeography of Neotropical Freshwater Fishes*, J. Albert, Ed. (University of California Press, 2011), pp. 59–67.

48. C. Hoorn, L. M. Boschman, T. Kukla, M. Sciumbata, P. Val, The Miocene wetland of western Amazonia and its role in Neotropical biogeography. *Bot. J. Linn. Soc.* **199**, 25–35 (2022).

49. C. Hoorn, F. P. Wesselingh, H. T. Steege, M. A. Bermudez, A. Mora, J. Sevink, I. Sanmartín, A. Sanchez-Meseguer, C. L. Anderson, J. P. Figueiredo, C. Jaramillo, D. Riff, F. R. Negri, H. Hooghiemstra, J. Lundberg, T. Stadler, T. Särkinen, A. Antonelli, Amazonia through time: Andean uplift, climate change, landscape evolution, and biodiversity. *Science* **330**, 927–931 (2010).

50. C. Hoorn, G. R. Bogotá-A, M. Romero-Baez, E. I. Lammertsma, S. G. A. Flantua, E. L. Dantas, R. Dino, D. A. do Carmo, F. Chemale, The Amazon at sea: Onset and stages of the Amazon River from a marine record, with special reference to Neogene plant turnover in the drainage basin. *Glob. Planet. Change* **153**, 51–65 (2017).

51. C. Hoorn, T. Kukla, G. Bogotá-Angel, E. van Soelen, C. González-Arango, F. P. Wesselingh, H. Vonhof, P. Val, G. Morcote-Rios, M. Roddaz, E. L. Dantas, R. V. Santos, J. S. S. Damsté, J.-K. Kim, R. J. Morley, Cyclic sediment deposition by orbital forcing in the Miocene wetland of western Amazonia? New insights from a multidisciplinary approach. *Glob. Planet. Change* **210**, 103717 (2022).

52. A. McDermott, A sea in the Amazon. *Proc. Natl. Acad. Sci.* **118**, e2102396118 (2021).

53. R. Bernal, C. D. Bacon, H. Balslev, C. Hoorn, S. J. Bourlat, H. Tuomisto, S. Salamanca, M. T. van Manen, I. Romero, P. Sepulchre, Could coastal plants in western Amazonia be relicts of past marine incursions? *J. Biogeogr.* **46**, 1749–1759 (2019).

54. C. Jaramillo, I. Romero, C. D'Apolito, G. Bayona, E. Duarte, S. Louwye, J. Escobar, J. Luque, J. D. Carrillo-Briceño, V. Zapata, A. Mora, S. Schouten, M. Zavada, G. Harrington, J. Ortiz, F. P. Wesselingh, Miocene flooding events of western Amazonia. *Sci. Adv.* **3**, e1601693 (2017).

55. P. O. Antoine, R. Salas-Gismondi, F. Pujos, M. Ganerød, L. Marivaux, Western Amazonia as a Hotspot of Mammalian Biodiversity Throughout the Cenozoic. *J. Mamm. Evol.* **24**, 5–17 (2017).

56. R. Salas-Gismondi, J. J. Flynn, P. Baby, J. V. Tejada-Lara, F. P. Wesselingh, P. O. Antoine, A miocene hyperdiverse crocodilian community reveals peculiar trophic dynamics in proto-Amazonian mega-wetlands. *Proc. Biol. Sci.* **282**, 20142490 (2015).

57. J. V. Tejada-Lara, R. Salas-Gismondi, F. Pujos, P. Baby, M. Benammi, S. Brusset, D. D. Franceschi, N. Espurt, M. Urbina, P. O. Antoine, Life in proto-Amazonia: Middle Miocene mammals from the Fitzcarrald Arch (Peruvian Amazonia). *Palaeontology* **58**, 341–378 (2015).

58. R. Salas-Gismondi, J. J. Flynn, P. Baby, J. V. Tejada-Lara, J. Claude, P. O. Antoine, A new 13 million year old gavialoid crocodylian from proto-amazonian mega-wetlands reveals parallel evolutionary trends in skull shape linked to longirostry. *PLOS ONE* **11**, e0152453 (2016).

59. W. Langston, Z. B. de Gasparini, "Crocodilians, Gryposuchus, and the South American gavials" in *Vertebrate Paleontology in the Neotropics. The Miocene Fauna of La Venta, Colombia*, R. F. Kay, R. H. Madden, R. L. Cifelli, J. J. Flynn, Eds. (Smithsonian Institution Press, 1997), pp. 113–154.

60. T. M. Scheyer, O. A. Aguilera, M. Delfino, D. C. Fortier, A. A. Carlini, R. Sánchez, J. D. Carrillo-Briceño, L. Quiroz, M. R. Sánchez-Villagra, Crocodylian diversity peak and extinction in the late Cenozoic of the northern Neotropics. *Nat. Commun.* **4**, 1907 (2013).

61. W. P. Maddison, D. R. Maddison, Mesquite: A modular system for evolutionary analysis. Version 2.75. 2011 (2015); http://mesquiteproject. org.

62. D. L. Swofford, PAUP*: phylogenetic analysis using parsimony, version 4.0b10. 21 Libro, doi: citeulike-article-id:2345226 (2003).

63. B. D. Boer, R. S. W. van de Wal, R. Bintanja, L. J. Lourens, E. Tuenter, Cenozoic global ice-volume and temperature simulations with 1-D ice-sheet models forced by benthic δ18O records. *Ann. Glaciol.* **51**, 23–33 (2010).

64. K. E. Campbell Jr, C. D. Frailey, L. Romero-Pittman, The Pan-Amazonian Ucayali Peneplain, late Neogene sedimentation in Amazonia, and the birth of the modern Amazon River system. *Palaeogeogr. Palaeoclimatol. Palaeoecol.* **239**, 166–219 (2006).

65. A. Traverse, *Paleopalynology* (Springer Science & Business Media, 2007), vol. 28.

66. C. Jaramillo, M. Rueda, A Morphological Electronic Database of Cretaceous-Tertiary and Extant pollen and spores from Northern South America, v.2022.

67. S. W. Punyasena, C. Jaramillo, F. De La Parra, Y. Du, Probabilistic correlation of single stratigraphic samples: A generalized approach for biostratigraphic data. *AAPG bulletin* **96**, 235–244 (2012).

68. D. Ochoa, C. Hoorn, C. Jaramillo, G. Bayona, M. Parra, F. De la Parra, The final phase of tropical lowland conditions in the axial zone of the Eastern Cordillera of Colombia: Evidence from three palynological records. *J. South Am. Earth Sci.* **39**, 157–169 (2012).

69. J. G. Mead, R. E. Fordyce, The Therian Skull: A Lexicon with Emphasis on the Odontocetes. Smithsonian Contributions to Zoology, 1–249 (2009).

70. F. C. Fraser, P. E. Purves, *Hearing in Cetaceans: Evolution of the Accessory Air Sacs and the Structure and Function of the Outer and Middle Ear in Recent Cetaceans* [Trustees of the British Museum (Natural History), 1960].

71. R. Salas-Gismondi, P. O. Antoine, P. Baby, S. Brusset, M. Benammi, N. Espurt, D. De Franceschi, F. Pujos, J. Tejada, M. Urbina, Middle Miocene crocodiles from the Fitzcarrald Arch, Amazonian Peru, *Cuadernos del Museo Geominero* **8**, 355–360 (2007).
